# 3D Protein structure prediction with genetic tabu search algorithm

**DOI:** 10.1186/1752-0509-4-S1-S6

**Published:** 2010-05-28

**Authors:** Xiaolong Zhang, Ting Wang, Huiping Luo, Jack Y Yang, Youping Deng, Jinshan Tang, Mary Qu Yang

**Affiliations:** 1 School of Computer Science and Technology, Wuhan University of Science and Technology, Wuhan, Hubei 430081, P.R. China; 2 Center for Research in Biological Systems, University of California at San Diego, La Jolla, California 92093-0043, USA; 3 School of Electrical and Computer Engineering, Purdue University, West Lafayette, Indiana 47907 USA; 4 Center for Computational Biology and Bioinformatics, Indiana University School of Medicine, Indiana University Purdue University, Indianapolis, Indiana 46202 USA; 5 International Society of Intelligent Biological Medicine and SpecPro Inc, 3909 Halls Ferry Road, Vicksburg, MS 39180, USA

## Abstract

**Background:**

Protein structure prediction (PSP) has important applications in different fields, such as drug design, disease prediction, and so on. In protein structure prediction, there are two important issues. The first one is the design of the structure model and the second one is the design of the optimization technology. Because of the complexity of the realistic protein structure, the structure model adopted in this paper is a simplified model, which is called off-lattice AB model. After the structure model is assumed, optimization technology is needed for searching the best conformation of a protein sequence based on the assumed structure model. However, PSP is an NP-hard problem even if the simplest model is assumed. Thus, many algorithms have been developed to solve the global optimization problem. In this paper, a hybrid algorithm, which combines genetic algorithm (GA) and tabu search (TS) algorithm, is developed to complete this task.

**Results:**

In order to develop an efficient optimization algorithm, several improved strategies are developed for the proposed genetic tabu search algorithm. The combined use of these strategies can improve the efficiency of the algorithm. In these strategies, tabu search introduced into the crossover and mutation operators can improve the local search capability, the adoption of variable population size strategy can maintain the diversity of the population, and the ranking selection strategy can improve the possibility of an individual with low energy value entering into next generation. Experiments are performed with Fibonacci sequences and real protein sequences. Experimental results show that the lowest energy obtained by the proposed GATS algorithm is lower than that obtained by previous methods.

**Conclusions:**

The hybrid algorithm has the advantages from both genetic algorithm and tabu search algorithm. It makes use of the advantage of multiple search points in genetic algorithm, and can overcome poor hill-climbing capability in the conventional genetic algorithm by using the flexible memory functions of TS. Compared with some previous algorithms, GATS algorithm has better performance in global optimization and can predict 3D protein structure more effectively.

## Background

Protein structure prediction is defined as the prediction of the tertiary structure of a protein by using its primary structure information [1]. Till now, it has become an important research topics in bioinformatics and it has important applications in medicine and other fields, such as drug design, prediction of diseases, and so on. Because of the complexity of the realistic protein structure, it is hard to determine the exact tri-dimensional structure from its sequence of amino acids [2]. Therefore, a lot of coarse structure models have been developed. The HP model is the most conventional one among them and has been widely used in protein structure prediction [3]. Different from the complex structure models, HP model only assumes two types of amino acids—hydrophobic (H) and hydrophilic (P) and the sequence of amino acids is assumed to be embedded in a lattice, which is used to discretize the space of conformations. For simplicity, the only interaction considered in HP model is the interaction between the nonadjacent but next-neighboured hydrophobic monomers [3], which is used to force the formation of a compact hydrophobic core as observed in real proteins [4].

Although simplified models have the capability of catching nontrivial aspects of the folding problem, the approximations involved are not really suitable [5]. The main reason lies in that local interactions are neglected in the simplified models. As is well known, local interactions might be important for the local structure of the chains [6] and no sequences with compact, well-defined native structures could be found if local interactions are neglected [5]. Therefore, many other models which consider local interactions have drawn a lot of attention and been proposed. The AB off-lattice model [7,8] is the one that could meet the aforementioned requirement. Currently, AB off-lattice model has been widely applied to protein structure prediction and many improved models  have been proposed based on the original model. In AB off-lattice model, two types of monomers are taken into consideration. The hydrophobic monomers are labelled by A while the hydrophilic ones are labelled by B. Different from HP model, the interactions considered in AB model include both sequence independent local interactions and the sequence dependent Lennard-Jones term that favours the formation of a hydrophobic core [7,8].

After a structure model is adopted, an important issue in PSP is to develop an optimization technology to find the best conformation of a protein sequence based on the assumed structure model. However, protein structure prediction (PSP) is an NP-hard problem even when the simplest models are assumed [9,10]. In order to tackle this issue, many heuristic approaches have been developed. In the past decades, researchers have developed many algorithms to solve the global optimization problem in protein folding structure prediction (PFSP). Genetic algorithm has been used for protein structure prediction for long time [11-17]. The reason why GAs are attractive is possibly due to their simplicity and efficiency in finding good solutions in large and complex search spaces [12,16]. It is well known that the combination of GA with local search strategies is particularly effective in PFP [2].

For example, the algorithm developed in [18] which is a hybrid scheme combining GA with simulated annealing algorithm, has much higher efficiency in searching for native states with off-lattice AB model than other methods. However, this method has a limitation that the searching time is too long, which affects its wide applications. In this paper we propose a novel hybrid approach for protein structure prediction. The proposed algorithm will combine genetic algorithm and tabu search algorithm to accurately search for the ground-state conformation of a given protein.

## Methods

### Off-lattice AB model

The off-lattice AB model was proposed in [7,8] and has been applied to protein structure prediction for decades. In off-lattice AB model, the monomers are linked by rigid unit-length bonds to form linear unoriented polymers in three-dimensional space. The energy functional for any n monomers chain is described as follows [8]:

                               (1)

where *θ_i_* (0≤*θ_i_*≤π) is the angle between two successive bond vectors. *r_ij_* is the distance between residues *i* and *j* with *i* <*j*. In three-dimensional space, *r_ij_* depends on both bond angle *θ* and torsional angle *β*. The constant *C*(*ξ_i_*,* ξ_j_*) is +1, +1/2, and -1/2 for *AA*, *BB* and *AB* pairs respectively [7].

In off-lattice AB model, the shape of an n-mer is determined by the (*n*-2) bond angles *θ*_1_,…,*θ_n_*_-2_, and the (*n*-3) torsional angles *β*_1_,…,*β_n_*_-3_. Therefore, the prediction of 3D folding structure problem of *n* monomers chain is equivalent to finding the optimal (*n*-2) bond angles and (*n*-3) torsional angles which minimize the energy functional *E* defined in equation (1).

### Improved strategies in genetic tabu search algorithm

Genetic algorithms [19] are adaptive heuristic search algorithms premised on the evolutionary ideas of natural selection and genetics, which select individuals by a fitness function. Individuals with higher fitness values have higher opportunity to generate the successors. Although genetic algorithms are widely used in optimization problems, they still need improvement for PSP. GA has two main disadvantages which affect their performance for PSP. One is the premature convergence and the other is the slow convergence rate. The premature convergence is mainly caused by the small variability in mutation strategy and the slow convergence rate results from heavy dependence on crossover strategy.

In order to overcome the disadvantages in GAs, we introduce tabu search (TS) [20] into the crossover and mutation operators in GAs to improve the local search capability. Tabu search [20] is a local neighborhood search algorithm which guides the next search direction by using flexible memory functions to record and choose the optimization process. The advantage of TS is the short searching time and the disadvantage is the low global search capability. Thus, the combination of GA and TS results in a hybrid algorithm which combines both of the advantages of the GA and TS [21].

The following five strategies are used in the proposed algorithm for protein structure prediction:

#### 1)  Chromosome encoding

Chromosome encoding is the way the individuals are represented and is very important because it affects the performance of a genetic algorithm. In the proposed algorithm, Cartesian coordinates are adopted to represent the individuals because of its simplicity. Let *h* be an individual. For an *n*-residue long chain, *h* can be expressed as (*θ*_1_,…,*θ_n_*_-2_, *β*_1_,…,*β_n_*_-3_), which concatenates the (*n*-2) bond angles and the (*n*-3) torsional angles. Cartesian coordinates of residue *i* in hypothesis *h*(*θ*_1_,…,*θ_n_*_-2_,*β*_1_,…,*β_n_*_-3_) is obtained as follows

(2)

The coordinates of the first few residues are (0,0,0), (0,1,0), and (cos(*θ*_1_),sin(*θ*_1_),0). Latter residues’ coordinates are all calculated on the base of the previous one’s coordinate.

#### 2) Variable population size

In genetic algorithm, with the difference between individuals get smaller and smaller after several rounds of evolution, premature convergence to poor solution will generally happen. Hence, the second strategy used in the proposed algorithm is to adopt variable population size. Variable population size strategy adopted by genetic algorithm can prevent premature convergence by increasing or decreasing the population size when the optimal energy is very close to the average value of the population [22].

#### 3)  Ranking selection

Ranking selection strategy is used in the proposed algorithm to select individuals from the current population into next generation. The main benefit of ranking selection lies in that it can bring the individuals with lower energy values into the next generation, and let the best individual obtained so far not be crossed out or damaged by mutation, and thus guarantee the convergence of the algorithm [22].With this strategy, whenever the hypothesis population has been updated, individuals are rearranged from minimum to maximum based on the energy values* E*(*h*) (obtained by (1)), and then the individuals in the front of the population will be made to have higher probability to be selected. The adoption of this strategy can make the best individuals enter into the next generation directly and avoid being operated by crossover and mutation operators.

#### 4)  Tabu search mutation (TSM)

**Figure 1:**
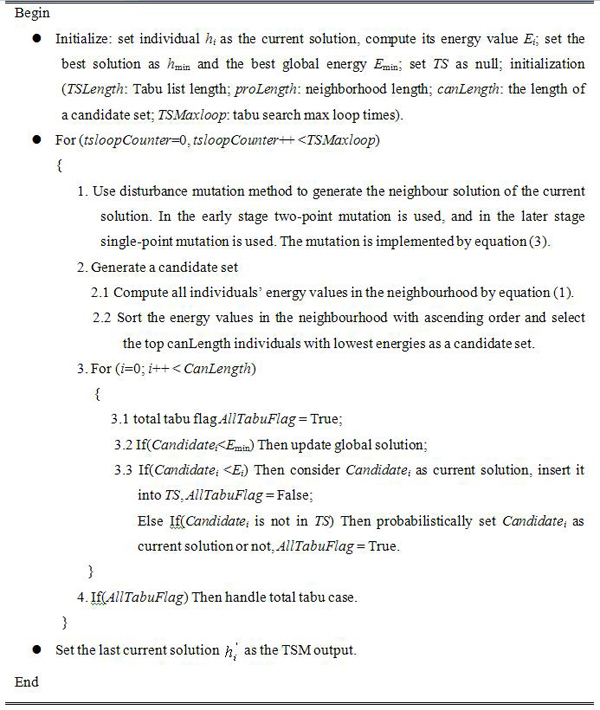
**TSM process** TSM is a search process. With this strategy, the potential energy functional in equation (1) is used as the evaluation function to compute offspring’s energy values, and then these offspring and their energies are combined with the tabu list to determine the output offspring.

In the proposed algorithm, the mutation operator adopted is tabu search mutation operator. Tabu search mutation operator is similar to the standard mutation operator except that TSM is a search process. With this strategy, the potential energy functional in equation (1) is used as the evaluation function to compute the offspring’s energy values, and then these offspring and their energy values are combined with the tabu list to determine the output offspring. Therefore, TSM can accept inferior solutions during the search process, and thus it has stronger hill-climbing capability than many other mutation operators [21,23]. TSM is composed of several steps (see Figure 1), which can be described as follows: Firstly, disturbance mutation method is used to generate neighbor solutions of the current solutions. In this processing, two mutation operations are used. The first mutation operation is a two-point mutation operation and is used in the early stage; the second mutation operation is a single-point mutation which is adopted in the later stage to raise the convergence speed. Disturbance mutation implementation is presented as follows. Let the *j *th parameter selected be  and the new parameter be, then we have

                                             (3)

where *r* is a random number between 0 and 1.  in term is used to assure large disturbance degree in the early search procedure (α tends to 0) to keep the diversity of the solutions, and small disturbance degree in the later search procedure (α tends to 1) to increase convergence rate and guarantee the algorithm to converge to a global optimum.  and  are defined as follows:

                                              (4)

                                            (5)

is used to ensure the diversity of the neighbour solutions, which is similar to [24]. *j* donates the location of the *j *th parameter in individual *h*, *n* is the parameter length of *h*. is the scale factor of parameter . Secondly, the individuals in the neighbor solutions are sorted by the energy values in ascending order and the lower energy individuals will be used to generate the candidate set. Finally, each solution in the candidate set will be determined to be the output of the TSM or not. This processing is based on two tabu lists as in [24]. The first tabu list is composed of a set of solution vectors and the second one is composed of a set of energy values of the corresponding solutions. The use of two tabu lists can let the algorithm avoid being trapped in local optima.

In order to determine whether a candidate solution is a tabu, we use the following criteria: let the energy value of the candidate solution  be *E*(*h*)(computed by (1)). If there is a solution  in tabu list *TS* which satisfies |*E*(*y*)-*E*(*h*)| ≤ *ψ* and ||*y*-*h*|| ≤ *η*, then the candidate solution *h* is thought as a tabu. In TSM, it is possible that total tabu [24] happens. When total tabu happens, all the solutions in the candidate set are forbidden and the next current solution can not be selected from the candidate set. In order to handle total tabu, when total tabu happens, we select the global best solution to generate mutation and the output solution will be set as current solution.

#### 5)  Tabu search recombination (TSR)

Another strategy used in the proposed algorithm is tabu search recombination. With this strategy, TSR records the fitness values of individuals in a tabu list and the fitness values of the offspring individual after crossover operation will be compared with some desired level and will be determined to be accepted by next generation or the tabu list. In this paper, the average fitness value of the population is considered as the desired level and the crossover strategy is random linear combination. Let  be one individual and  be the other individual of the crossover couple, be the offspring individual after crossover operation, random linear combination is described by [25]

				                    (6)

where  is the individual with minimum energy so far.

In order to avoid close match, before the crossover operation, TSR uses the following way to select the individuals as the crossover individual couple into cross pool: it will first sort the individuals by their energy values in ascending order, and then the individuals with the furthest distance are selected for crossover operation. After the crossover offspring  is obtained, its fitness value is compared with the desired level. The comparison is as follows: if the fitness value of the offspring is better than the desired value, the offspring will be set free, and accepted by next generation. If the fitness value of the offspring is worse than the desired value and is not in tabu list, the offspring will also be accepted. However, if the offspring is in the tabu list, TSR will select one parent with better fitness value to go into the next generation. In TSR, the use of tabu list keeps the diversity among individuals and avoids premature convergence.

### Genetic tabu search algorithm

Genetic algorithm and tabu search algorithm have their own advantages and disadvantages, thus the development of a scheme which keeps the advantages while overcomes the disadvantages of each algorithm can provide efficient search for protein structure prediction. GATS, a hybrid algorithm, satisfies this requirement. For example, GATS makes use of the advantages of multiple search points in GA and can overcome the poor hill-climbing capability by using the flexible memory functions of TS.

**Figure 2:**
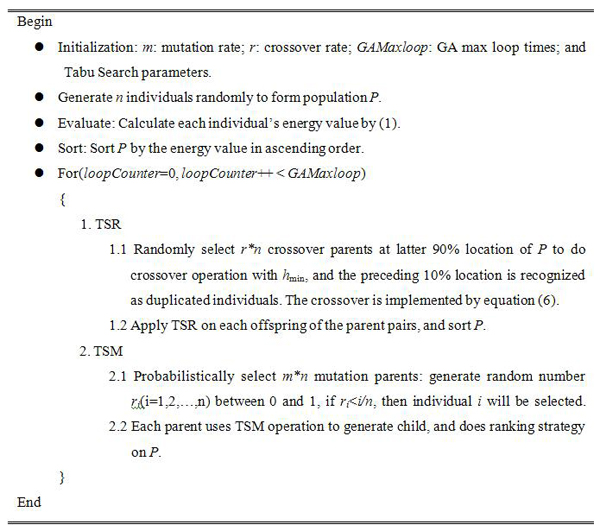
**Genetic tabu search algorithm** The hybrid algorithm combines genetic algorithm and tabu search algorithm and can deal with multi-extremum and multi-parameter problems.

The basic idea of the proposed GATS is described in Figure 2. The search algorithm starts with the initialization of parameters by some appropriate values. Then, the population *P* with individuals  is generated randomly, and equation (1) is used to obtain the energy values. After that, the individuals are sorted by the energy values from minimum to maximum and at the same time, the minimal solution and the minimal energy are saved as *h*_min_ and *E*_min_ respectively. During the search process, population *P* is handled by TSR and TSM by turns. When TSR handles the population, it will select r*n parents from the latter 90% locations to perform crossover operation with *h*_min_, and the preceding 10% locations are recognized as duplicated individuals. The offspring will be considered whether are accepted based on the current tabu list. When TSM handles the population, m*n mutation parents will be selected probabilistically, and each parent uses TSM operation to generate offspring. Whenever the population *P* is updated, individuals will be rearranged to be from minimal to maximal by the energy values. Finally, the hypothesis *h*_min_ and minimum energy *E*_min_ will be used as the optimal values at the end of algorithm.

## Results and discussion

### Results for Fibonacci sequences

In this section, we describe our experiments by using Fibonacci sequences to test the efficiency of the proposed GATS. A Fibonacci sequence is defined recursively by

*S*_0_ = *A, S*_1_ = *B, S_i_*_+1_ * *S_i_*. (7)

where * is the concatenation operator. Some examples of Fibonacci sequences are *S*_2_ = *AB*, *S*_3_ = *BAB*, *S*_4_ = *ABBAB*, etc. For comparison, we used the same Fibonacci sequences as those used in [24,26-28].

GATS was implemented by C++ in Windows XP. The parameters in the algorithm were obtained by experiments and they were set as follows: self-adjustable population scale was set to be in the range of 100~500, the crossover rate was set to be 0.88, the mutation rate was set to be in the range of 0.012~0.025, self-adjustable tabu list length was set to be in the range of 7~14, neighbourhood set length was set to be in the range of 30~50, candidate set length was set to be in the range of 5~6. The minimal energy values () obtained by GATS on the three-dimensional off-lattice AB model are listed in Table 1. For comparison, we also list the minimal energy values obtained by the Simulated Annealing (SA) [28], the energy landscape paving minimizer (ELP) [26], the conformational space annealing (CSA) [27], and the tabu search algorithm (TS) [24] respectively. The corresponding bond angles and the torsional angles at the global minima are shown in Table 2.

**Table 1:** Lowest energies for Fibonacci sequences obtained by the previous algorithms and the proposed GATS algorithm

N	SEQUENCES					
13	ABBABBABABBAB	-4.9746	-4.967	-4.9746	-6.5687	-6.9539
21	BABABBABABBABBABABBAB	-12.0617	-12.316	-12.3266	-13.4151	-14.7974
34	ABBABBABABBABBABABBABABBABBABABBAB	-23.0441	-25.476	-25.5113	-27.9903	-27.9897
55	BABABBABABBABBABABBABABBABBABABBABBABABBABABBABBABABBAB	-38.1977	-42.428	-42.3418	-41.5098	-42.4746

**Table 2:** The (n-2) bond angles and (n-3) torsional angles at the global minimum energies of four Fibonacci sequences by GATS

N	bond angles(*θ*_1_,…,*θ_n_*_-2_)	torsional angles (*β*_1_,…,*β_n_*_-3_)
13	0.11611, -1.44697, 0.32017, 0.01576,0.50275, -0.86862, 0.12051, -0.58378,-0.45150, -0.89987, -0.00446	0.00102, -0.67737, -2.14380, 2.65948, -0.12802, 2.11709, 0.24980, 1.82580, -2.99295, 1.46672
21	-0.08637, -1.24407, -0.02388, 0.71560, 0.20713, 1.77167, 0.25962, -0.31786, 1.57686, -0.95041, 0.04461, 0.03119, 0.54498, -0.82860, 0.37745, -0.62814, -0.98493, -0.38993, -0.23650	2.97004, 0.58008, 2.15906, -2.64461, 0.40657, -2.00601, 2.47012, 2.63691, -1.09615, 3.11625, -1.56743, -0.03975, -2.96520, 0.06719, -1.86684, -0.32537, 1.10542, -0.70935
34	0.23573, -1.43459, 0.29165, -1.84341, 0.14631, -1.61093, -0.35038, 1.67799, 0.17042, 1.60574, -0.40332, -1.26662, -0.27629, 1.20980, 0.21014, 1.29636, 0.00999, -1.69196, -0.35294, 0.72788, -0.62924, 0.17575, -0.34728, -0.97787, -0.16688, -0.91012, -0.07292, 1.03188, 2.48730, -0.16685, -0.69786, 0.68108	-0.14964, 0.63562, 0.75934, -2.74033, 0.28633, -2.61899, -2.91781, -0.44565, -0.12498, -0.84551, -2.50502, 2.53977, -2.54137, 1.87607, -2.82187, 2.47530, 2.87212, -2.10783, 0.15378, -2.88144, 1.93040, 0.43629, 2.41795, 0.72296, -0.58509, 1.13745, -2.92674, 0.68888, -2.37150, -0.79336, 2.98078
55	0.57994, -0.54747, -1.63058, 0.32512, 1.29499, 0.94198, 0.15639, 0.54547, 0.51204, 2.42050, 0.42994, 0.04798, 0.53466, 0.72372, -2.84018, -0.25987, -0.88420, 0.50741, 0.31571, -0.38491, -0.36698, -0.85173, 0.13171, -0.28528, 1.24401, -0.44344, 0.32826, -0.71533, -0.52747, -0.08801, -0.44238, -0.05707, -0.08495, -0.62277, 0.07570, -0.90285, -0.24254, 0.16364, -0.47504, -0.50923, -0.37872, 0.57320, 1.66339, 0.32637, -0.74187, 0.43684, -0.15112, 1.46664, 0.34051, -0.72797, -0.07620, -0.73615, -0.79086	-2.62178, 2.15436, 2.79679, -2.21273, -2.62632, -0.37956, -2.18578, -1.26221, -0.51001, 2.21957, -2.55211, -1.00242, -2.74164, -2.46270, -2.53201, 2.51849, 0.55237, -1.22255, 0.70861, -1.09153, 0.34246, 2.12777, 0.25911, 0.39082, -2.89463, -0.93970, 3.02711, -1.82971, -1.76602, -2.81629, 1.66725, 1.77810, 0.81533, 2.01598, -0.19887, 1.65355, 1.11533, 0.46418, -1.38864, 0.55938, -1.12062, 0.29809, 1.89867, -2.71331, -2.06007, -1.76112, 0.24818, -1.91570, -2.01395, -0.43327, 0.97151, -0.82385

From table 1, we can find that the lowest energy value obtained by the proposed GATS is smaller than those obtained by SA, ELP and CSA for all the four Fibonacci sequences, and smaller than that obtained by TS for the sequences with lengths 13, 21, 55. Although the lowest energy value obtained by GATS is not as low as that obtained by TS for the sequence with length 34, it is smaller than that obtained by TS for the sequences with lengths 55, which shows that GATS has better performance for long sequence.

**Figure 3:**
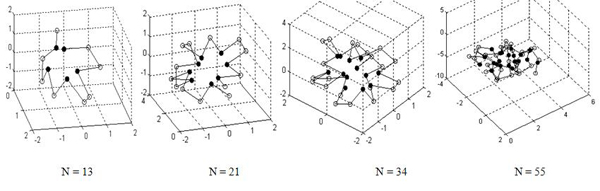
**The lowest energy conformations for the four Fibonacci sequences obtained by GATS algorithm** Solid dots indicate hydrophobic monomers *A*, and open dots indicate hydrophilic monomers *B*.

The lowest-energy ground configurations of Fibonacci sequences listed in Table 1 are presented in Figure 3. In Figure 3, the solid dots indicate the hydrophobic monomers *A* while the empty dots indicate the hydrophilic monomers *B*. Figure 3 shows that all the conformations form single compact hydrophobic cores surrounded by hydrophilic residues, which is observed in real proteins. The results verify that it is reasonable to use AB model with Fibonacci sequences in three dimensions to mimic the real protein.

### Results for real protein sequences

In this section, we describe the experimental results using real protein sequences. The real protein sequences used in our experiments were downloaded from the website: http://pdbbeta.rcsb.org/pdb/Welcome.do. For comparison, we used the same three protein sequences as those used in [29]. The PDB ID of the three protein sequences are 1BXL, 1EDP and 1AGT, respectively. In the experiments, the same K-D method used in [29,30] were adopted to distinguish the hydrophobic monomers from the hydrophilic ones, where *I, V, L, P, C, M, A, G* are considered to be hydrophobic while *D, E, F, H, K, N, Q, R, S, T, W, Y* are hydrophilic. Because there are few papers dealing with the real protein structure prediction issue using off-lattice AB model, we only compared our experimental results with the results in [29].

**Figure 4:**
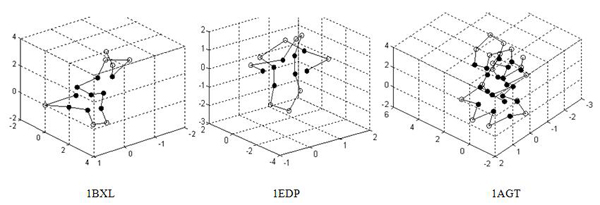
**The lowest energy conformations for the three real protein sequences obtained by GATS algorithm** Solid dots indicate hydrophobic monomers *I, V, L, P, C, M, A, G,* and open dots indicate hydrophilic monomers *D, E, F, H, K, N, Q, R, S, T, W, Y*.

The experimental results for the real proteins are presented in Table 3, and the corresponding lowest protein landscapes obtained by our GATS are shown in Figure 4. Table 3 shows that the minimal energy values obtained by the proposed GATS are lower than those obtained by TS in [29], especially for long sequences. The results demonstrate that GATS is much more efficient than TS in protein folding structure prediction using AB off-lattice model. From Figure 4, we find that all the configurations have also formed a hydrophobic core, surrounded by hydrophilic residues. However, the hydrophobic core of 1AGT, which is the longest among the three real proteins, seems not to be compact enough. This may indicate that the performance of the coarse simplified AB off-lattice model is not effective enough for the prediction of the structure for long protein sequences.

**Table 3:** Minimum energies for three real proteins obtained by TS and GATS algorithm using off-lattice AB model in three dimensions.

PDB ID	SEQUENCES		
1BXL	GQVGRQLAIIGDDINR	-15.7164	-15.8246
1EDP	CSCSSLMDKECVYFCHL	-12.8392	-13.7769
1AGT	GVPINVSCTGSPQCIKPCKDQGMRFGKCMNRKCHCTPK	-44.2656	-46.0842

The PDB ID is unique identifier of a protein in the database, representing its amino acid sequences

## Conclusions

A hybrid algorithm that combines genetic algorithm and tabu search algorithm is developed for 3-D protein structure prediction using off-lattice AB model. The proposed algorithm can deal with multi-extremum and multi-parameter problems. In the proposed algorithm, different strategies are adopted to make the proposed algorithm have different advantages. For examples, the variable population size strategy can keep the diversity of the population, and TSM strategy makes it possible to accept poor solution as the current solution and thus makes the algorithm have better hill-climbing capability and stronger local searching capability than many other mutation operators. In addition, TSR strategy can limit the frequency that the offsprings with the same fitness appear, and thus can also keep the diversity of the population and avoid premature convergence of the algorithm. Compared with the previous algorithms, GATS has stronger capability of global searching. In the future work, we will improve the algorithm and make it more effective for long protein sequence prediction using multi-core computing platforms [31].

## Competing interests

The authors declare that they have no competing interests.

## Authors' contributions

XZ designed the algorithm and analyzed the experimental results. TW participated in the implementation of algorithm, and did the experiments with the given data. HL took part in the implementation of the algorithm and data processing. XZ, TW and HL wrote the original version of the paper. JT and YD helped rewriting the paper based on the original version. JYY and MQY contributed to the development of the algorithm, and provided many useful insights on protein modeling. All authors agreed on the content of the paper.
